# Association Between *RSK2* and Clinical Indexes of Primary Breast Cancer: A Meta-Analysis Based on mRNA Microarray Data

**DOI:** 10.3389/fgene.2021.770134

**Published:** 2021-11-01

**Authors:** Kun Zheng, Shuo Yao, Wei Yao, Qianxia Li, Yali Wang, Lili Zhang, Xiuqiong Chen, Huihua Xiong, Xianglin Yuan, Yihua Wang, Yanmei Zou, Hua Xiong

**Affiliations:** ^1^ Department of Oncology, Tongji Hospital, Tongji Medical College, Huazhong University of Science and Technology, Wuhan, China; ^2^ Biological Sciences, Faculty of Environmental and Life Sciences, University of Southampton, Southampton, United Kingdom; ^3^ Institute for Life Sciences, University of Southampton, Southampton, United Kingdom

**Keywords:** ribosomal protein S6 kinase, 90kDa, polypeptide 3 (RSK2), breast cancer, molecular subtype, microarray, prognostic value, biomarkers

## Abstract

**Background:** Although ribosomal protein S6 kinases, 90 kDa, polypeptide 3 (*RSK2*, *RPS6KA3*) has been reported to play an important role in cancer cell proliferation, invasion, and migration, including breast cancer, its clinical implication in primary breast cancer patients is not well understood, and there were not many studies to explore the relationship between *RSK2* and breast cancer on a clinical level.

**Methods:** A systematic series matrix file search uploaded from January 1, 2008 to November 31, 2017 was undertaken using ArrayExpress and Gene Expression Omnibus (GEO) databases. Search filters were breast cancer, RNA assay, and array assay. Files eligible for inclusion met the following criteria: a) sample capacity is over 100, b) tumor sample comes from unselected patient’s primary breast tumor tissue, and c) expression of *RSK2* and any clinical parameters of patients were available from the files. We use median as the cutoff value to assess the association between the expression of *RSK2* and the clinical indexes of breast cancer patients.

**Finding:** The meta-analysis identified 13 series matrix files from GEO database involving 3,122 samples that come from patients’ primary breast cancer tissue or normal tissue. The expression of *RSK2* in tumor tissues is lower than that in normal tissues [odds ratio (OR), 0.54; 95% credible interval (CI), 0.44–0.67; Cochran’s *Q* test *p* = 0.14; *I* ^2^ = 41.7%]. Patients with a high expression of *RSK2* showed more favorable overall survival [hazard ratio (HR), 0.71; 95% CI, 0.49–0.94; Cochran’s *Q* test *p* = 0.95; *I* ^2^ = 0.0%] and less potential of distant metastasis (OR, 0.59; 95% CI, 0.41–0.87; Cochran’s *Q* test *p* = 0.88; *I* ^2^ = 0.0%) and lymph node infiltration (OR, 0.81; 95% CI, 0.65–0.998; Cochran’s *Q* test *p* = 0.09; *I* ^2^ = 42.8%). Besides, the expression of *RSK2* in luminal breast cancer is lower than Cochran’s *Q* test *p* = 0.06; *I* ^2^ = 63.5%). *RSK2* overexpression corresponded with higher histological grade (OR, 1.329; 95% CI, 1.03–1.721; Cochran’s *Q* test *p* = 0.69; *I* ^2^ = 0.0%). *RSK2* expression is also associated with estrogen receptor (ER) and age.

**Conclusion:** The meta-analysis provides evidence that *RSK2* is a potential biomarker in breast cancer patients. The expression of *RSK2* is distinctive in different intrinsic subtypes of breast cancer, indicating that it may play an important role in specific breast cancer. Further study is needed to uncover the mechanism of *RSK2* in breast cancer.

**Systematic Review Registration:** (website), identifier (registration number).

## Introduction

Breast cancer is the most frequent cancer among women. Data from the World Health Organization shows that breast cancer impacts over 1.5 million women each year and also causes the greatest number of cancer-related deaths among women. In 2015, 570,000 women died from breast cancer—that is, approximately 15% of all cancer deaths among women. Although there has been a breakthrough in the treatment and prevention of breast cancer in the past few years, leading to the 5-years relative survival rate rising to 90%, the majority of breast cancer patients with distant metastasis succumb to cancer progression within 5 years (Siegel et al., 2018). Also, breast cancer is more than one single disease. Several molecular subtypes of breast cancer have been classified depending on their molecular characteristics (Perou et al., 2000), and each individual subtype corresponds to a different underlying biology, survival rate, and response to therapy (Prat et al., 2015; Nielsen et al., 2017). Therefore, the identification of biomarkers to screen high-risk patients, predict breast prognostic outcomes, and provide new therapeutic targets for specific breast cancer is urgently needed.

*RSK2*, ribosomal protein S6 kinase, 90 kDa, polypeptide 3, belongs to *RSK* serine/threonine kinase family and is a downstream of the mitogen-activated protein kinase (MAPK) pathway (Zhao et al., 2016). *RSK* is unique among serine–threonine kinases in that it contains two functional kinase domains: an N-terminal kinase that phosphorylates the substrates of *RSK* and a C-terminal kinase involved in the activation mechanism of *RSK* (Frödin and Gammeltoft, 1999). *RSK* isoforms are activated by virtually all extracellular signaling molecules including growth factors, peptide hormones, neurotransmitters, and environmental stresses (Arul and Cho, 2013). It has been demonstrated that *RSK2* plays an important role in cancer cell proliferation, invasion, and migration, including breast cancer (Yoo et al., 2019; Guo and Kong, 2021).

In previous studies, it has been reported that *RSK2* expression is different between breast cancer tissue and normal breast tissue and varies among different subtypes or histological grades of breast cancer. Some studies suggested that *RSK2* overexpression is correlated with basal-like breast cancer and higher histological grade, and *RSK2* mRNA is associated with poor survival in breast cancer patients who had not received chemotherapy (Stratford et al., 2012; Zhao et al., 2016). A protein downstream of *RSK2* named Y-box binding protein-1(*YB-1*) was reported to transform human mammary epithelial cells in the development of basal-like breast cancer (Davies et al., 2014). Another study indicated that *RSK2* activation status positively correlates with patient response to anti-estrogen hormonal therapies and is required for estrogen receptor+ (ER+) breast cancer tumorigenesis (Clark et al., 2001). Several drug trials illustrated that by suppressing *RSK2* expression, the metastasis of human epidermal growth factor receptor 2+ (*HER2*+) breast cancer was repressed (Mao et al., 2016), the ability of migration and invasion of lung cancer cell was inhibited (Lee et al., 2015), and the carcinogenesis of ultraviolet radiation-induced skin cancer was prevented (Yao et al., 2014). These prompt us to investigate whether *RSK2* might be a potential biomarker that can act as a promising biomarker of breast cancer or a novel therapy target for a specific subtype of breast cancer. However, *RSK2* expression is rarely associated with clinical practice, which drives us to investigate the association between *RSK2* expression and clinical parameters and prognosis of breast cancer patients.

In the case of very limited clinical studies of *RSK2*, we performed a meta-analysis using public electronic databases ArrayExpress (Parkinson et al., 2007) and Gene Expression Omnibus (GEO) (Clough and Barrett, 2016) to summarize and evaluate the clinical significance of *RSK2* in breast cancer patients and in order to explore the possibility of *RSK2* expression as a predictive marker of clinicopathological parameters and prognosis in primary breast cancer, so as to screen high-risk patients or to provide new targets and directions for the treatment of breast cancer patients with specific molecular subtypes.

## Methods

### Literature Search

We conducted a search of a series matrix files in the electronic database ArrayExpress (ArrayExpress, 2017) uploaded from January 1, 2008 to November 1, 2017 using the search filter “breast cancer,” “*Homo sapiens*,” “RNA assay,” “array assay,” and “all assay.” We also conducted a search of a series matrix files in the GEO database (NCBI, 2017) uploaded from January 1, 2008 to November 1, 2017 using the search filter “breast cancer,” “*Homo sapiens*,” “series,” and “expression profiling by array.” A total of 207 and 227 expressions by array dataset were listed in the ArrayExpress and GEO databases, respectively. Relevant literatures were found in GEO database using the GSE ID.

### Inclusion and Exclusion Criteria

This meta-analysis collects data from primary breast cancer patient’s tumor tissue to assess the relationship of *RSK2* expression and the clinical parameters of breast cancer patients, such as clinicopathological features and prognostic factors. Inclusion criteria are as follows: a) patients in the study have not been selected, or the selection had no effect on clinical indicators given the *RSK2* expression might be different among different subtypes of breast cancer and different clinical status of patients; b) the sample size in each file is greater than 100 and comes from the same study; c) sample comes from breast cancer patients’ primary tumor tissue or normal tissue; d) the expression of *RSK2* was efficient and available from the series matrix file; and e) any of a patient’s clinicopathological features and prognostic factors can be extracted from the file. When samples in a file are duplicated with samples in another file, we selected the larger or qualified one. The exclusion criteria are as follows: a) samples were gathered from different studies; b) the original study could not be found; c) the sample size in the document is inconsistent with the number of patients in the study, and the replicated samples could not be found in the file; and d) the file was ineligible after invalid samples were removed. Files were filtered by two researchers separately; the disagreement was resolved through discussion. The eligible files are listed on Table 1.

**TABLE 1:** Studies included in the meta-analysis.

First author	GSE ID	Platform	Sample (N)	Year	Country or area	Duration (months)	Quality score
Calabrò et al. (2009)	GSE10510	GPL6486	152	2009	Germany	36	9
Haakensen et al. (2010)	GSE18672	GPL6848	143	2010	Norway	24	7
Enerly et al. (2011)	GSE19783	GPL6480	115	2011	Norway	44	8
Kao et al. (2011)	GSE20685	GPL570	327	2011	Taiwan	168	9
Terunuma et al. (2014)	GSE39004	GPL6244	108	2014	USA	127	8
Clarke et al. (2013)	GSE42568	GPL570	121	2013	Ireland	60	7
Gruosso et al. (2016)	GSE45827	GPL570	155	2016	France	NA	7
Lu et al. (2008)	GSE5460	GPL570	129	2008	USA	NA	8
Tofigh et al. (2014)	GSE58644	GPL6244	317	2014	Switzerland	NA	9
Huang et al. (2015)	GSE59595	GPL17581	175	2015	Italy	12	9
Chanrion et al. (2008)	GSE9893	GPL5049	155	2008	France	156	9
Li et al. (2010)	GSE19615	GPL570	115	2010	USA	NA	7
Wang et al. (2015)	GSE93601	GPL22920	1,110	2015	USA	324	9

### Data Extraction

For observational studies, the Newcastle-Ottawa Quality Assessment Scale (NOS) was employed for assessing the quality of these studies. All data was abstracted by using a standardized data collection form, with information recorded as follows: first author’s name, publication year, country of origin, number of cases and controls, detection method, GSE ID, and platform of detection. Each sample’s *RSK2* expression and corresponding clinicopathological features and prognostic factors were extracted from the series matrix file, including age, tissue, ER status, progesterone receptor (PR) status, *HER2* status, lymph node infiltration, histological grade, TNM stage, tumor size, tumor type, metastasis, intrinsic subtype (by PAM50), overall survival time (OS), disease-free survival time (DFS), relapse-free survival time (RFS), and relevant status of the patient. Samples with incomplete information or data described above were removed.

Data extraction is conducted by two researchers, respectively, and disagreements were resolved by discussion.

### Statistical Analysis

Statistical analysis was conducted by the guidelines proposed by the Meta-Analysis of Observational Studies group (Stroup et al., 2000). Median was used as the cutoff value to determine the level of *RSK2* expression because there was no suitable cutoff value to help us distinguish the expression status of *RSK2*. Odds ratio (OR) was employed for evaluating the association between *RSK2* expression and clinicopathological features. Hazard ratio (HR) and 95% credible interval (CI) were appraised to assess the association between *RSK2* expression and prognostic indicators, including OS, DFS, and RFS by using IBM SPSS Statistics 24. Heterogeneity of the OR and HR was calculated by using the Cochran’s *Q* and *I* ^2^ test. A random-effect model was applied when *p* < 0.1 or *I* ^2^ > 50%. When heterogeneity was absent, a fixed-effect model was employed. Begg’s rank correlation method and Egger’s weighted regression methods were employed to assess publication bias. STATA software package (version 12.0) was used to calculate pooled ORs, HRs, and corresponding 95% CI; all *p* values were two tailed.

Given the limited prognostic information of specific breast cancer patients in those GSE files, we used the Kaplan–Meier Plotter (Hou et al., 2017; Kaplan-Meier plotter), an online database including gene expression data and clinical data, to assess the prognostic value of *RSK2* in breast cancer. The patient samples were divided into two cohorts according to the median expression of the gene (high vs. low expression).

## Results

### Search Results

**FIGURE 1:**
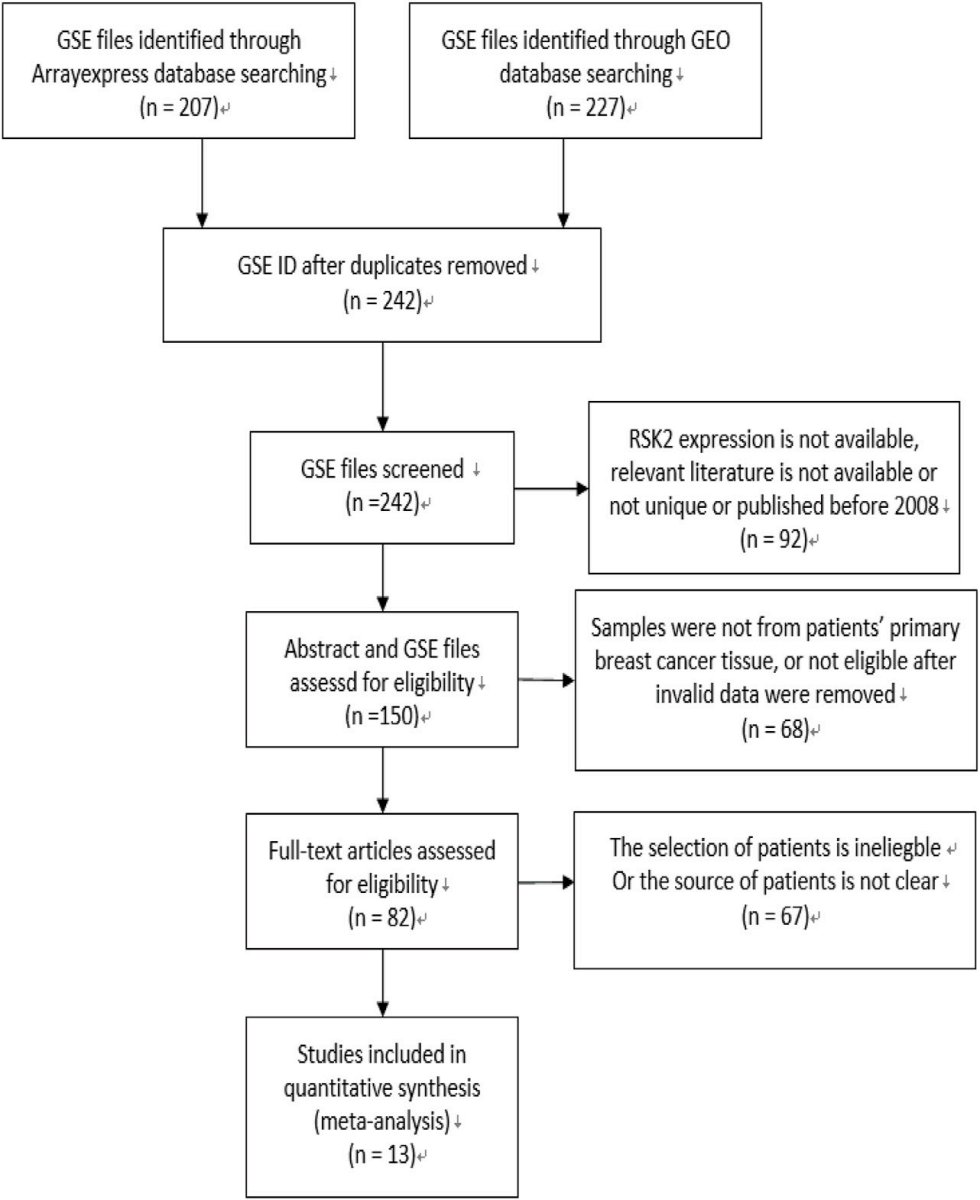
Flow diagram of literature selection. The search and selection process for the expression of *RSK2* of breast cancer patients and the number of eligible studies.

The flow diagram for the recognition of eligible studies is presented in Figure 1. There were 207 and 227 GSE files identified from the ArrayExpress database and GEO database, respectively. After duplicates were removed, *RSK2* expression, abstract, and full text were checked, and 13 GSE files from 13 independent studies involving 3,122 patients were identified by our search strategy. The features of the 13 studies are listed in Table 1. Ineligible samples and data were removed, such as samples from cell, blood, or distant metastasis. When sample was detected multiple times, the one with the highest *RSK2* expression was selected. Histological grades Ⅰ and Ⅱ were grouped as low-grade disease, and Ⅲ was grouped as high-grade disease. Clinical stages Ⅰ and Ⅱ were grouped as early-stage disease, and III and IV were grouped as late-stage disease. Tumors larger than 2 cm were grouped as large tumors, and the rest were grouped as small tumors. Patients were divided into high-age group and low-age group, with 55 years old as the cutoff value. Clinical stage of GSE20685 was not available, which we estimated depending on the T, N, and M stage shown in the GSE file using the NCCN guidelines of breast cancer (version 2. 2011). Data in GSE39004 were only used for comparing *RSK2* expression between normal tissues and tumor tissues, because its tumor sample size is not large enough.

### *RSK2* Expression in Breast Tumor Tissues was Lower Than That in Normal Breast Tissue and Enriched in Basal-Like Breast Cancer

**FIGURE 2:**
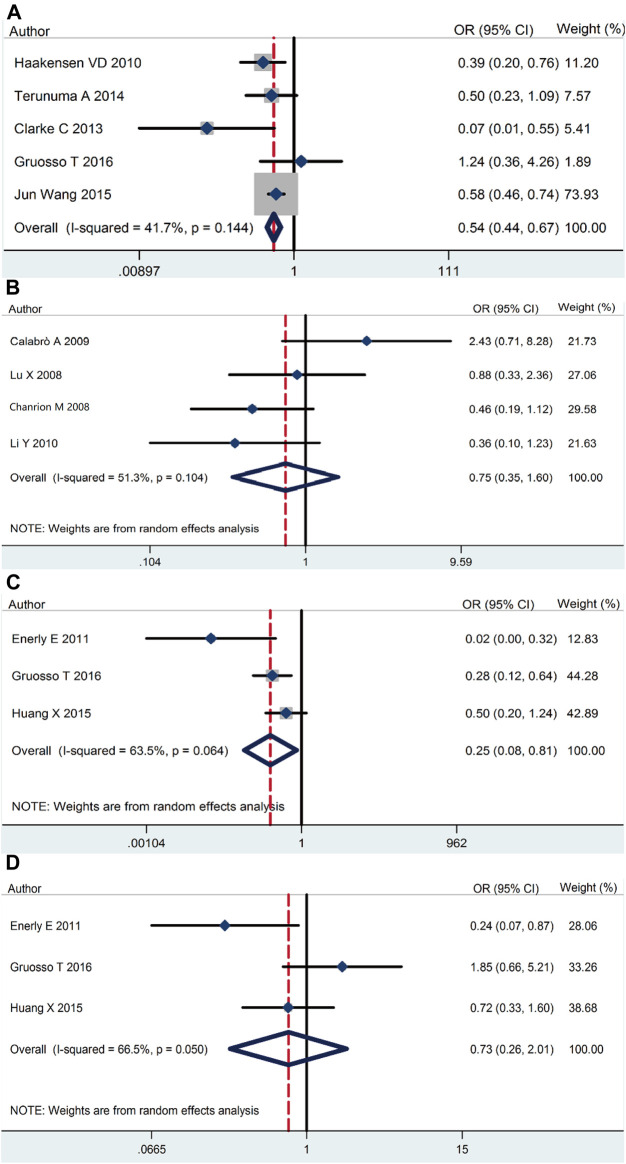
*RSK2* expression among different types of breast tumor tissues as well as normal breast tissues. **(A)** *RSK2* expression in breast cancer tissue compared with normal tissue. **(B)** The association between *RSK2* expression and the ductal breast cancer relative to the lobular breast cancer. **(C)** *RSK2* expression in luminal breast cancer compared with basal-like breast cancer. **(D)** *RSK2* expression in luminal A breast cancer compared with luminal B breast cancer.

Our meta-analysis demonstrated that *RSK2* expression in breast cancer tissue was lower than that in normal tissue (pooled OR = 0.54, 95% CI: 0.44–0.67, Cochran’s *Q* test *p* = 0.14, *I* ^2^ = 41.7%) (Figure 2A). There was no statistically significant difference between ductal carcinoma and lobular carcinoma (pooled OR = 0.75, 95% CI: 0.35–1.60, Cochran’s *Q* test *p* = 0.104, *I* ^2^ = 51.3%) (Figure 2B). The relationship of *RSK2* expression and molecular subtype was analyzed in our meta-analysis. The expression of *RSK2* was obviously different between the luminal subtype and basal subtype of breast cancer (pooled OR = 0.25, 95% CI: 0.08–0.80, Cochran’s *Q* test *p* = 0.06, *I* ^2^ = 63.5%) (Figure 2C), and no distinctive *RSK2* expression was found between luminal A and luminal B subtypes of breast cancer (pooled OR = 0.73, 95% CI: 0.26–2.01, Cochran’s *Q* test *p* = 0.05, *I* ^2^ = 66.5%) (Figure 2D).

### *RSK2* Expression is Negatively Correlated With ER Status

**FIGURE 3:**
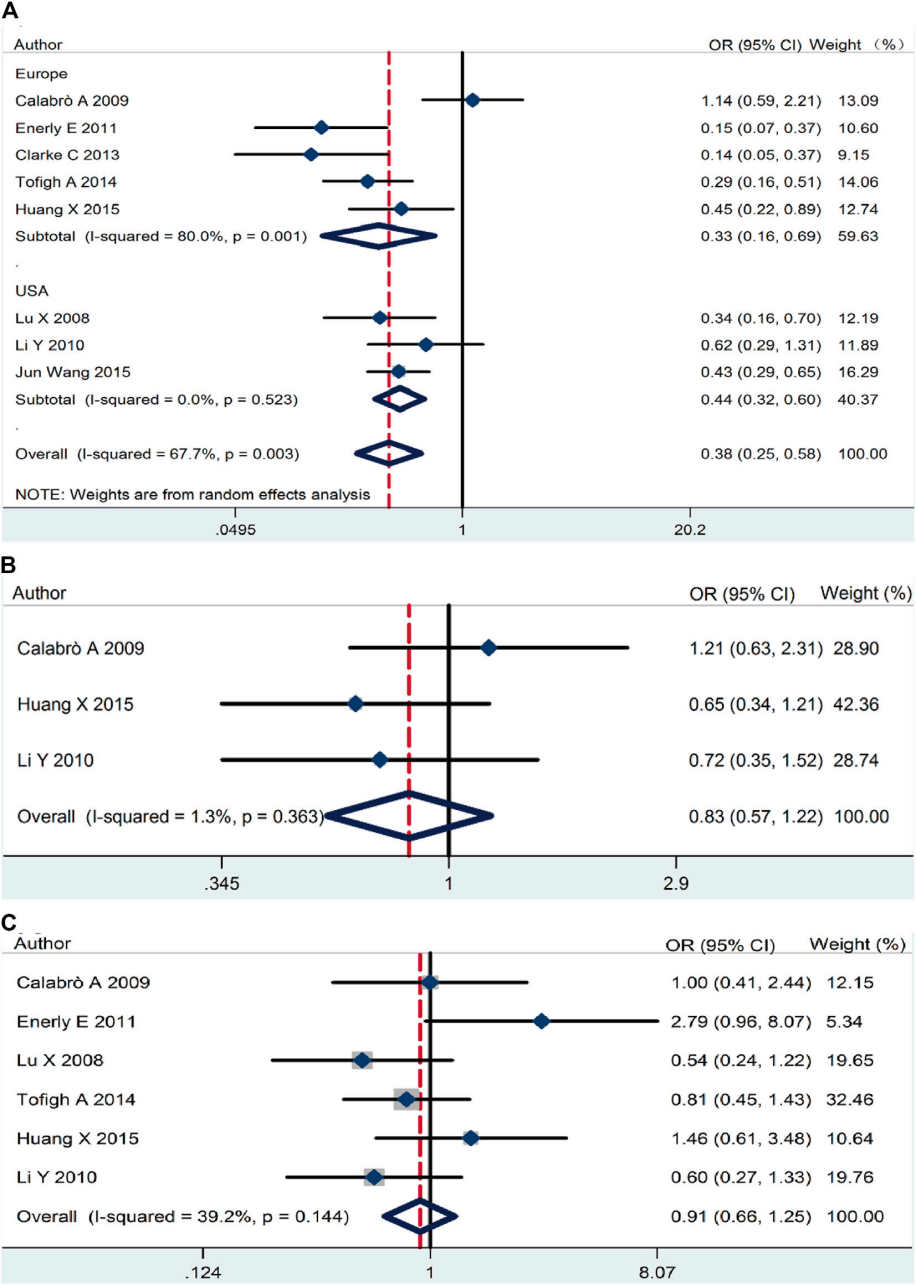
The association between *RSK2* expression and the three main breast cancer biomarkers. **(A)** The association between *RSK2* expression and estrogen receptor (ER) status. **(B)** The association between *RSK2* expression and progesterone receptor (PR) status. **(C)** The association between *RSK2* expression and *HER2* status.

On the basic data obtained, we analyzed the relationship between *RSK2* expression and the three main breast cancer biomarkers. *RSK2* expression was inversely correlated with ER expression (pooled OR = 0.38, 95% CI: 0.25–0.58, Cochran’s *Q* test *p* = 0.009, *I* ^2^ = 67.7%) (Figure 3A). There was no statistically significant relationship between PR expression (pooled OR = 0.83, 95% CI: 0.57–1.22, Cochran’s *Q* test *p* = 0.36, *I* ^2^ = 1.3%) (Figure 3B) and *HER2* expression (pooled OR = 0.91, 95% CI: 0.66–1.25, Cochran’s *Q* test *p* = 0.14, *I* ^2^ = 39.2%) (Figure 3C).

### *RSK2* Expression Effects on Breast Cancer Progression

**FIGURE 4:**
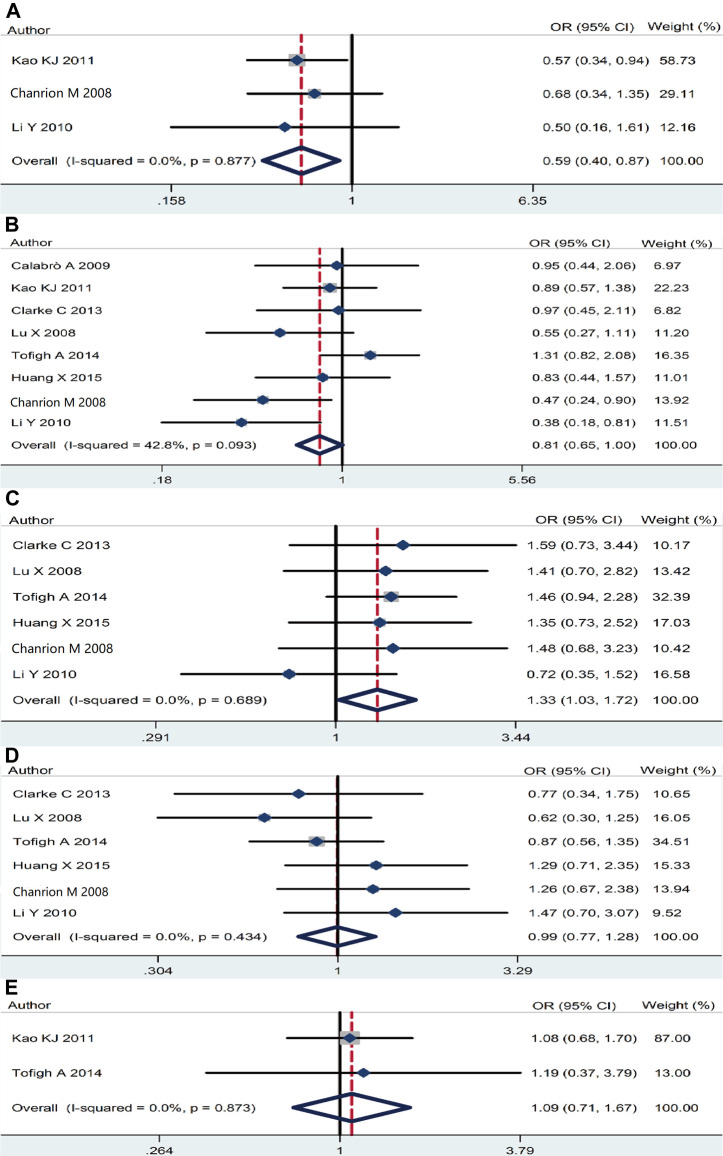
The association between *RSK2* expression and other clinicopathological characters. **(A)** The association between *RSK2* expression and distant metastasis. **(B)** The association between *RSK2* expression and lymph node infiltration. **(C)** The association between *RSK2* expression and histological grade. **(D)** The association between *RSK2* expression and tumor size. **(E)** The association between *RSK2* expression and clinical stage of breast cancer.

The association of *RSK2* expression with breast cancer clinicopathological features was also analyzed. High *RSK2* expression reduced the possibility of distant metastasis (pooled OR = 0.59, 95% CI: 0.41–0.87, Cochran’s *Q* test *p* = 0.88, *I* ^2^ = 0.0%) (Figure 4A) and lymph node metastasis (pooled OR = 0.81, 95% CI: 0.65–0.998, Cochran’s *Q* test *p* = 0.09, *I* ^2^ = 42.8%) (Figure 4B). The overexpression of *RSK2* positively correlated with histological grade (pooled OR = 1.33, 95% CI: 1.03–1.72, Cochran’s *Q* test *p* = 0.69, *I* ^2^ = 0.0%) (Figure 4C). However, *RSK2* expression has no statistically significant relationship with other biological characters of breast cancer, including tumor size (pooled OR = 0.995, 95% CI: 0.77–1.28, Cochran’s *Q* test *p* = 0.43, *I* ^2^ = 0.0%) (Figure 4D) and clinical stage (pooled OR = 1.09, 95% CI: 0.71–1.67, Cochran’s *Q* test *p* = 0.87, *I* ^2^ = 0.0%) (Figure 4E).

### High *RSK2* Expression is Indicative of Longer OS in Breast Cancer Patients

**FIGURE 5:**
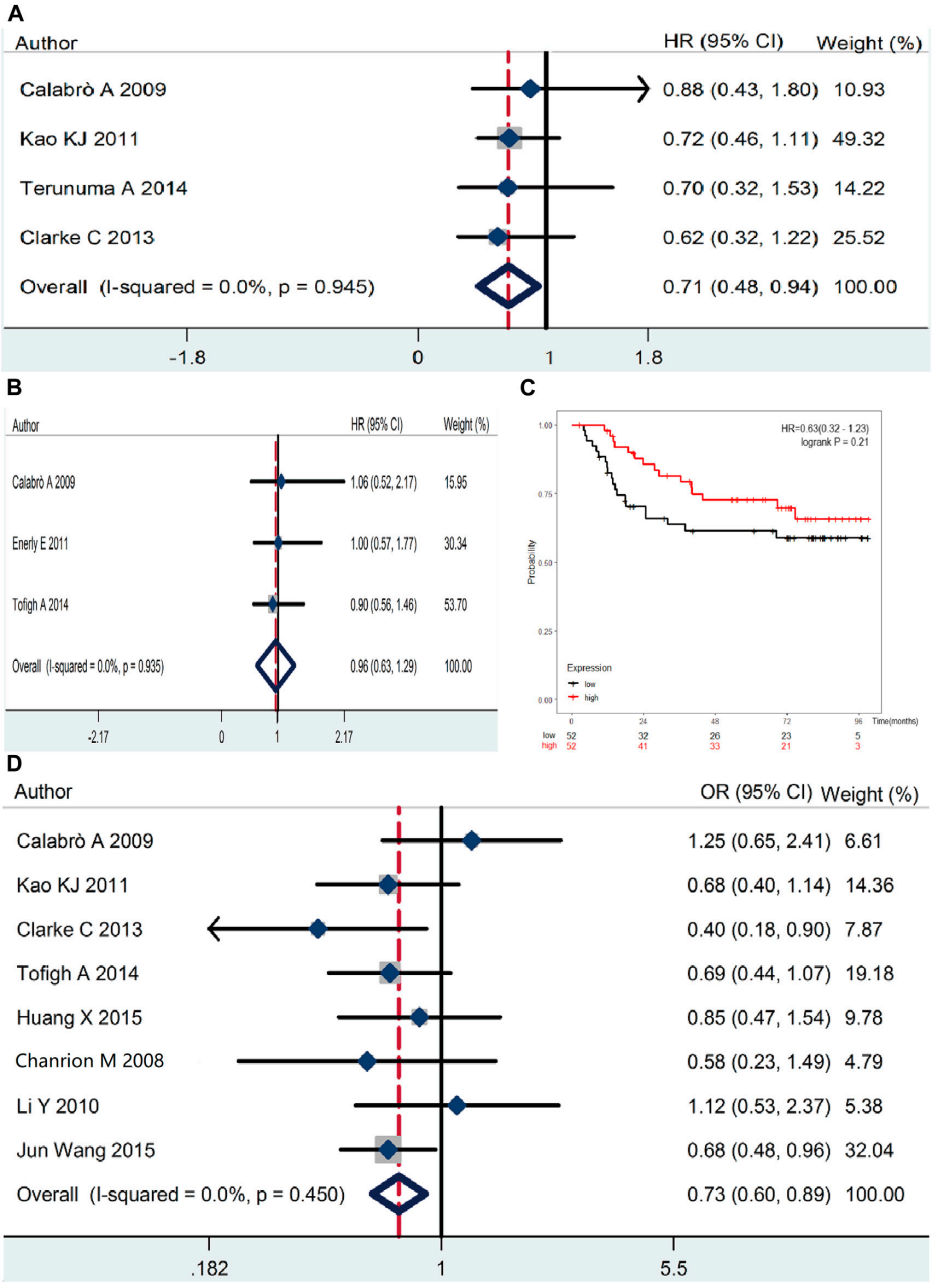
The association between *RSK2* expression and survival outcome of breast cancer patients. **(A)** The association between *RSK2* expression and breast cancer overall survival (OS). **(B)** The association between *RSK2* expression and breast cancer disease-free survival (DFS). **(C)** The association between *RSK2* expression and age of breast cancer patients.

We evaluated the association between *RSK2* expression level and survival outcome of breast cancer patients. The results indicate that *RSK2* overexpression was statistically associated with the OS rate of breast cancer patients (pooled HR = 0.71, 95% CI: 0.48–0.94, Cochran’s *Q* test *p* = 0.95, *I* ^2^ = 0.0%) (Figure 5A), while there was no significant relationship between *RSK2* expression and DFS (pooled HR = 0.96, 95% CI: 0.63–1.29, Cochran’s *Q* test *p* = 0.94, *I* ^2^ = 0.0%) (Figure 5B). Only one GSE file (GSE42568) involving 104 patients has the data of RFS, and there was no statistical significance between them (HR = 0.62, 95% CI: 0.32–1.22, log rank test *p* = 0.21) (Figure 5C). We did not find a significant difference in survival outcome between basal-like breast cancer and luminal breast cancer for the limited sample capacity and accessible data.

### *RSK2* Expression is Negatively Correlated With the Age of Breast Cancer Patients

We also recorded the corresponding age of every sample and grouped them into low-age group and high-age group with 55 years old as the cutoff value, which was randomly selected. Interestingly, we found that *RSK2* expression is lower in the high-age group (pooled OR = 0.73, 95% CI: 0.60–0.89, Cochran’s *Q* test *p* = 0.45, *I* ^2^ = 0.0%) (Figure 5D). In order to investigate whether *RSK2* expression decreased with age, we calculated the relationship between *RSK2* expression and age involving 508 normal tissue samples in GSE93601, and no statistically significant correlation was found (OR = 0.95, 95% CI: 0.66–1.36). The status of p53 was available in GSE19783, which involves 110 patients, indicating there was a positive relationship between *RSK2* and P53 mutation (OR = 4.09, 95% CI: 1.74–9.22, Cochran’s *Q* test *p* = 0.0007).

### Prognostic Value of Various *RSK2* Among Different Molecular Subtypes of Breast Cancer

**FIGURE 6:**
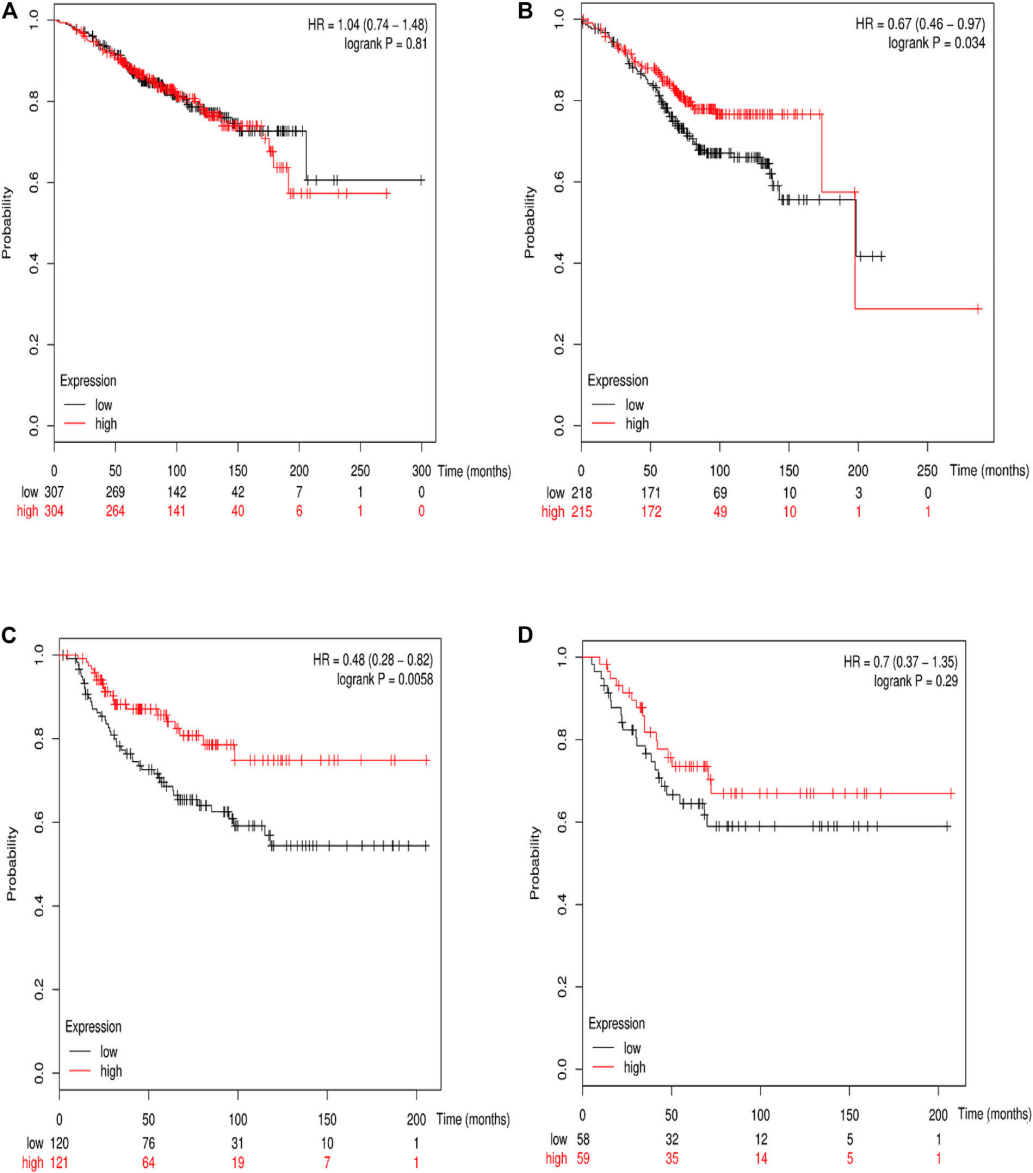
The association between *RSK2* expression and OS in different molecular subtypes of breast cancer. **(A)** The association between *RSK2* expression and OS in luminal A breast cancer. **(B)** The association between *RSK2* expression and OS in luminal B breast cancer. **(C)** The association between *RSK2* expression and OS in basal-like breast cancer. **(D)** The association between *RSK2* expression and OS in *HER2*+ breast cancer.

**FIGURE 7:**
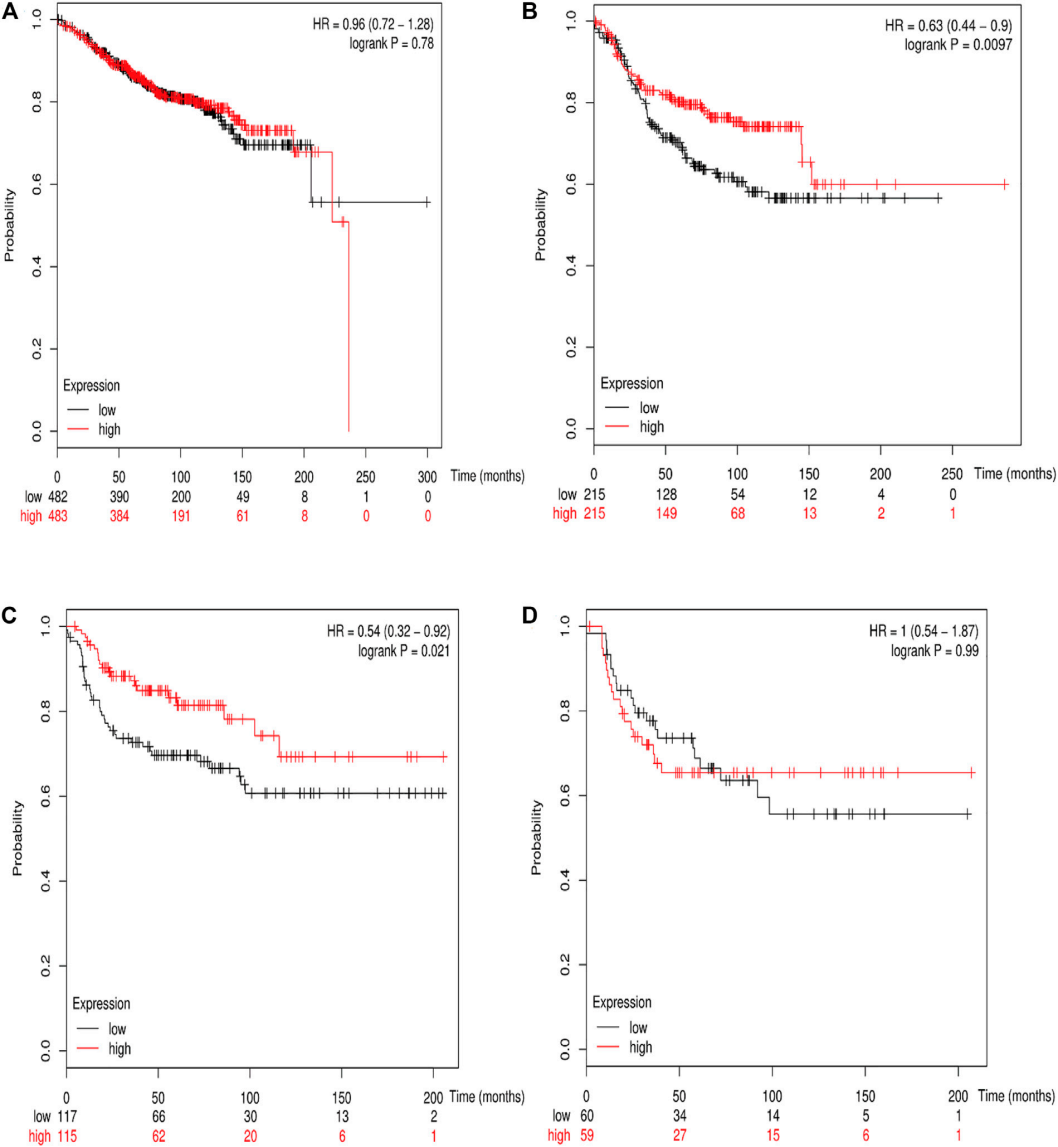
The association between *RSK2* expression and distant metastasis-free survival (DMFS) in different molecular subtypes of breast cancer. **(A)** The association between *RSK2* expression and DMFS in luminal A breast cancer. **(B)** The association between *RSK2* expression and DMFS in luminal B breast cancer. **(C)** The association between *RSK2* expression and DMFS in basal-like breast cancer. **(D)** The association between *RSK2* expression and DMFS in *HER2*+ breast cancer.

**FIGURE 8:**
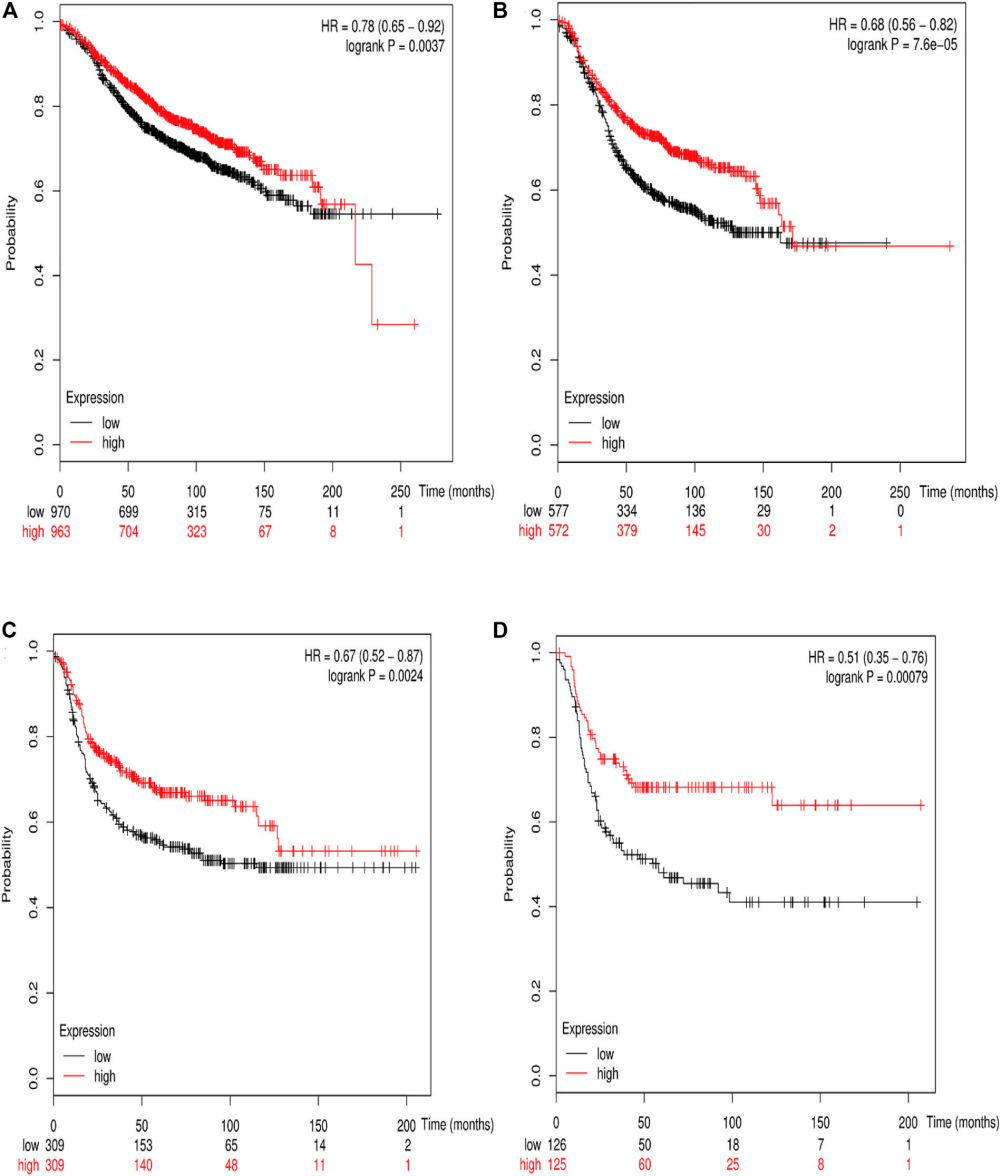
The association between *RSK2* expression and relapse-free survival (RFS) in different molecular subtypes of breast cancer. **(A)** The association between *RSK2* expression and RFS in luminal A breast cancer. **(B)** The association between *RSK2* expression and RFS in luminal B breast cancer. **(C)** The association between *RSK2* expression and RFS in basal-like breast cancer. **(D)** The association between *RSK2* expression and RFS in *HER2*+ breast cancer.

The online database Kaplan–Meier was employed to evaluate the impact of *RSK2* expression on the prognostic outcome in different molecular subtypes of breast cancer, indicating that elevated *RSK2* expression predicts a favorable OS (luminal A breast cancer: HR = 1.04, 95% CI: 0.74–1.48, log rank *p* = 0.81; luminal B breast cancer: HR = 0.67, 95% CI: 0.46–0.97, log rank *p* = 0.034; basal-like breast cancer: HR = 0.48, 95% CI: 0.28–0.82, log rank *p* = 0.006; *HER2*+ breast cancer: HR = 0.7, 95% CI: 0.37–1.35, log rank *p* = 0.29) (Figure 6) and distant metastasis-free survival (DMFS) (luminal A breast cancer: HR = 0.96, 95% CI: 0.72–1.28, log rank *p* = 0.78; luminal B breast cancer: HR = 0.63, 95% CI: 0.44–0.9, log rank *p* = 0.009; basal-like breast cancer: HR = 0.54, 95% CI: 0.32–0.92, log rank *p* = 0.021; *HER2*+ breast cancer: HR = 1, 95% CI: 0.54–1.87, log rank *p* = 0.99) (Figure 7) in basal-like and luminal B breast cancer, but not in luminal A and *HER2*+ breast cancer. The overexpression of *RSK2* predicts a favorable prognostic value of RFS (Figure 8) in all those subtypes of breast cancer (luminal A breast cancer: HR = 0.78, 95% CI: 0.65–0.92, log rank *p* = 0.004; luminal B breast cancer: HR = 0.68, 95% CI: 0.56–0.82, log rank *p* < 0.001; basal-like breast cancer: HR = 0.67, 95% CI: 0.52–0.87, log rank *p* = 0.002; *HER2*+ breast cancer: HR = 0.51, 95% CI: 0.35–0.76, log rank *p* < 0.001).

The sample capacity is very large in GSE93601, which may have a great impact on the statistical results. We reanalyzed the data after removing the data from GSE93061 and got the same result as the previous one.

### Publication Bias

Publication bias statistics were obtained using the Begg’s test and Egger’s test, which did not indicate any significant publication bias (Table 2).

**TABLE 2:** Publication bias tested by Egger’s test and Begg’s test.

	Egger’s test *P*	Begg’s test *P*
Normal tissue and tumor tissue	0.476	0.806
ER status	0.412	0.266
PR status	0.936	1
*HER2* status	0.264	0.260
Lymph node infiltration	0.149	0.536
Tumor size	0.890	1
Histological grade	0.582	1
Basal-like and luminal breast cancer	0.325	1
Luminal A and B breast cancer	0.764	1
Distant metastasis	0.866	1
Lobular and ductal breast cancer	0.615	1
Age of patient	0.776	0.902
Overall survival	0.892	1
Disease-free survival	0.222	0.296

ER, estrogen receptor; PR, progesterone receptor.

## Discussion

*RSK2* is an X-linked dominant gene and acts as a modulator of craniofacial development, and the mutation of *RSK2* was responsible for Coffin–Lowry syndrome (Laugel-Haushalter et al., 2014). It is generally believed that *RSK2* plays an important role in the tumorigenesis, migration, invasion, cell proliferation, and response to stress (Sulzmaier and Ramos, 2013; Laugel-Haushalter et al., 2014; Alesi et al., 2016). Precisely measuring the prognostic value of *RSK2* may help to guide individual therapies for breast cancer patients. Our meta-analysis takes advantage of a public electronic database to evaluate the association between the abundance of *RSK2* mRNA and the clinical parameters of breast cancer patients for the first time. Although it is not possible to draw conclusions about causality, these findings suggest that *RSK2* is a potential biomarker in breast cancer, especially in a specific subtype of breast cancer, and might provide new perspective of the interaction between *RSK2* and breast cancer.

From our research, *RSK2* expression was overexpressed in basal-like breast cancer and higher histological grade breast cancer and negatively correlated with estrogen receptor. These results corresponded with previous studies that suggest that *RSK2* expression is highest in basal-like breast cancer and those with the highest histological grade (Stratford et al., 2012; Zhao et al., 2016). A protein downstream of *RSK2*, namely *YB-1*, transforms human mammary epithelial cells through chromatin remodeling leading to the development of basal-like breast cancer (Davies et al., 2014). Inactivating *YB-1* can depress tumor-initiating cells of basal-like breast cancer. A study suggested that ER-α physically interacts with *RKS2*, resulting in the accumulation of *RSK2* in nuclear sequestration, and *RSK2* can promote neoplastic transformation and facilitate metastatic tumor growth of ER+ breast cancer (Ludwik et al., 2018), but there was no explanation for *RSK2* expression negatively correlating with ER status. However, there is no obvious statistical significance between *RSK2* expression and progesterone receptor on the basic data. Luminal A breast cancer is an ER-positive breast cancer with a lower histological grade, while luminal B breast cancer is an ER-positive breast cancer with a higher histological grade. Although *RSK2* is overexpressed in higher histological grade breast tumor, there is no obvious distinction between luminal A and luminal B breast cancer from our data. It may be partly due to the limited sample size of luminal A and luminal B breast cancer, and the distinction of *RSK2* expression was not large enough between them.

There were some unexpected results based on our data. It is generally believed that the expression of *RSK2* in cancer tissue is higher than that in normal tissue, and reducing the expression of *RSK2* can prevent tumorigenesis, tumor cell growth, and ability of migration and invasion (Lee et al., 2013; Yao et al., 2014; Mao et al., 2016; Zhao et al., 2016). However, based on our data, the contrary result was obtained. Furthermore, no evidence indicates that the immunohistochemistry outcome of *RSK2* was different from that of mRNA microarray assay. The overexpression of *RSK2* alone or *RSK2* combined with other biomarkers indicates a poor prognostic outcome was reported. For example, it has been shown that targeting *RSK2* with specific inhibitors or small interfering RNAs remarkably inhibits the growth and renewal of tumor-initiating cells in triple-negative breast cancer (TNBC) and that *RSK2* promotes migration through the ERK/MEK pathway (Stratford et al., 2012). In addition, Czaplinska et al. found that fibroblast growth factor receptor 2 (*FGFR2*) can form an indirect complex with *RSK2*, which may be involved in the progression of breast cancer and lead to poor prognosis in breast cancer patients (Czaplinska et al., 2016).

Based on the data collected from microarray, the OS of breast cancer patients is higher in *RSK2* high-expression patients than that in *RSK2* low-expression patients, and with the increase of *RSK2* expression, the potential of distant metastasis and lymph node infiltration decreased. Moreover, it is strange that the expression of *RSK2* is highest in basal-like breast cancer (TNBC), which is defined by the absence of the three main breast cancer biomarkers—i.e., a lack of expression of ER and PR and a lack of amplification or overexpression of *HER2—*and cooperates with poor prognosis and high risk of distant metastasis (Carey et al., 2010), but the OS, the potential of distant metastasis, and the lymph node metastases were more favorable in the *RSK2* high-expression group in the basic data from microarray. No relevant study was available to help us understand the mechanism under the paradoxical phenomenon. We hypothesize that *RSK2* plays a different role in different subtypes of breast cancer. We did not find a significant difference in survival outcomes between basal-like breast cancer and luminal breast cancer for the limited sample capacity and accessible data. In reference to the result from the online Kaplan–Meier Plotter, the overexpression of *RSK2* predicts more favorable prognostic value of RFS in all subtypes of breast cancer. As for the OS and DMFS, only basal-like and luminal B breast cancer patients were able to benefit from *RSK2* overexpression.

We found that *RSK2* expression is negatively correlated with the age of breast cancer patients for the first time, but there is no such relationship in normal tissue. There is also no statistically significant difference in *RSK2* expression between the early-stage group and late-stage group of breast cancer patients. It was reported that *RSK2* is sequestered in stress granules, which can aid cell survival in response to environmental stress by acting as sites of translational repression, and facilitates stress granule assembly to repress translation and to enhance cell survival (Eisinger-Mathason et al., 2008). The body’s response to stress decreases with age and may provide a possible explanation for the phenomenon.

Heterogeneity tests are an essential part of a meta-analysis. In this study, minor heterogeneities were observed with respect to OS, DFS, tumor size, clinical stage, and histological grade; however, there were substantial heterogeneities with respect to ER status, *HER2* status, lymph node infiltration, and different subtypes of breast cancer. This unbalanced phenomenon could partly result from the detection method and accuracy of ER status, PR status, and *HER2* status being different from each other, and the data completeness obtained from GSE files was not identical. Three GSE files have efficient PR status, and the heterogeneity was not obvious among them, while the other three GSE files have identified the molecular subtype of breast cancer, which shows a significant heterogeneity. Patients from different areas may respond to the heterogeneities. There were no heterogeneities in United States patients, while the main heterogeneity of ER status and *HER2* status originates from different European countries when we conducted a subgroup analysis. Another significant heterogeneity was likely due to the detection platform. Publication bias is worth considering in a meta-analysis. In this study, there was no significant publication bias based on the Egger’s and Begg’s test.

There are still some limitations in this meta-analysis. First of all, the relevant studies and complete available data were limited, and the available clinical parameter is not homogenous among those matrix files. Secondly, the detection platform, method, and accuracy of hormone receptors are different among these studies. Thirdly, the therapy level and method are distinctive, and we cannot eliminate their effect. Lastly, we cannot ignore the publication bias. Some data are still unavailable.

For further verification, we could download the mRNA expression data and corresponding clinical information of breast cancer patients from other databases as a validation cohort to verify the relationship between the *RSK2* expression level and the clinicopathological features as well as the prognosis of patients. In terms of experimental validation, future researchers could modify the expression of *RSK2* in different breast cancer cell lines and then perform various *in vitro* and mice xenografts *in vivo* trials to observe the effects of altering *RSK2* expression on the proliferation, apoptosis, cell cycle, metastasis, and invasion capabilities of breast cancer cells. Furthermore, the prognostic significance of *RSK2* could also be verified by measuring the protein expression level of *RSK2* by flow cytometry, western blotting, and immunohistochemistry staining on tumor and paracancerous normal tissues of breast cancer patients in conjunction with clinical information analysis. The strategies above could help to confirm the reliability of our meta-analysis findings based on *RSK2* mRNA expression and prognosis.

In conclusion, our meta-analysis was the first study that used microarray assay to research the association between *RSK2* expression and clinicopathological features and prognostic factors of primary breast cancer patients. Although some results corresponded with previous studies and some results were opposite to previous studies, both of them indicated *RSK2* is a promising biomarker of breast cancer. This study provides a new research direction and area of *RSK2*, while more experimental studies and elaborate research are needed to uncover the sealed mechanism of *RSK2* in breast cancer.

## Data Availability

The original contributions presented in the study are included in the article/Supplementary Material, further inquiries can be directed to the corresponding authors.

## Abbreviations

CI, credible interval; DFS, disease-free survival time; DMFS, distant metastasis-free survival; ER, estrogen receptor; FGFR2, fibroblast growth factor receptor 2; GEO, gene expression omnibus; HER2, human epidermal growth factor receptor 2; HR, hazard ratio; MAPK, mitogen-activated protein kinase; NOS, newcastle-ottawa quality assessment scale; OR, odds ratio; OS, overall survival; PR, progesterone receptor; RFS, relapse-free survival time; RSK2/RPS6KA3, ribosomal protein S6 kinases, 90 kDa, polypeptide 3; TNBC, triple-negative breast cancer; YB-1, Y-box binding protein-1.
